# Surgical training does not affect operative time and outcome in total knee arthroplasty

**DOI:** 10.1371/journal.pone.0197850

**Published:** 2018-06-01

**Authors:** Markus Weber, Michael Worlicek, Florian Voellner, Michael Woerner, Achim Benditz, Daniela Weber, Joachim Grifka, Tobias Renkawitz

**Affiliations:** 1 Department of Orthopaedic Surgery, Regensburg University, Medical Center, Bad Abbach, Germany; 2 Department of Hematology and Oncology, Regensburg University, Medical Center, Regensburg, Germany; Harvard Medical School, UNITED STATES

## Abstract

Training the next generation of orthopaedic surgeons in total knee arthroplasty (TKA) is crucial, but might affect operative time and outcome. We hypothesized that the learning curve of residents in TKA has an impact on (1) operative time, (2) complication rates and (3) early postoperative outcome. In a retrospective analysis of 738 primary TKAs from our institutional joint registry, operative time, complication rates, patient-reported outcome measures (EQ-5D, WOMAC) within the first year and responder rates for positive outcome as defined by the OMERACT-OARSI criteria were compared between trainee and senior surgeons differentiating between conventional and navigated TKA. Mean operative time was 69.5±18.5min for trainees compared to 77.3±25.8min for senior surgeons (95%CI of the difference 1.5–13.9min, p = 0.02) in conventional TKA and 80.4±22.1min to 84.1±27.6min (95%CI of the difference -0.9–8.2min, p = 0.12) for navigated TKA, respectively. Intraoperative fracture (p≥0.36), thrombosis (p≥0.90), neurological deficits (p≥0.90) and infection rates (p≥0.28) were comparably low in both groups. Patient-reported outcome measures one year after TKA were similar for trainee and senior surgeons with EQ-5D 0.83±0.17 to 0.80±0.21 (p = 0.25) and WOMAC 74.85±18.60 to 72.77±20.12 (p = 0.44) for conventional TKA and EQ-5D 0.80±0.20 to 0.82±0.18 (p = 0.23) and WOMAC 72.71±18.52 to 75.77±17.78 (p = 0.07) for navigated TKA, respectively. Similarly, responder rates for positive outcome were comparable between trainees and senior surgeons (90.7% versus 87.0% p = 0.39 for conventional TKA, 88.7% versus 89.4% p = 0.80 for navigated TKA). Supervised TKA is a safe procedure during the learning curve of young orthopaedic surgeons.

## Introduction

Total knee arthroplasty (TKA) is a widely performed and successful procedure in orthopaedic surgery [1]. For primary TKA, an increase of 673% to 3.48 million procedures is estimated in the United States by 2030 [2]. The additional demand for arthroplasty surgeons is faced with a high number of expected retirements threatening access to TKA and thus leading to a potential supply side crisis [3]. These bleak prospects emphasize the high need of training young surgeons in performing TKAs.

In contrast, the initiation of national joint registries, quality networks and online platforms urges hospitals to avoid patient dissatisfaction and complication rates and prolonged operative time in their statistical reports [4, 5]. Furthermore, the growing socioeconomic pressure for time efficient surgery additionally interferes with the opportunity for young surgeons to train operative procedures. However, the subject of outcome and complication rates after TKA performed by trainees is still a matter of debate [6–9].

The use of imageless computer navigation for TKA was developed in the 1990s to improve leg alignment and produce more reliable results [10, 11]. Over the last decade, this technology spread throughout the world and has become more and more popular. Besides the pros [10, 12] and cons [13, 14] of navigated TKA stated in orthopaedic literature, imageless navigation harbors the potential of an educational tool for young surgeons. The real-time calculation of the system allows the surgeon both to evaluate bony resections prior to execution and to control their accuracy immediately afterwards [14]. This might represent a potential benefit for a trainee within his learning curve for TKA.

In the current single center study of 738 primary TKAs, we aimed to compare operative time, complication rates and early postoperative outcome within the first year after TKA between trainees and senior surgeons at a university medical center differentiating between navigated and conventional TKA.

## Patients and methods

A retrospective analysis of the institutional joint registry was performed. The local Ethics Commission waived approval due to the retrospective study design. A power calculation was performed for investigation of the primary endpoint operative time. The corresponding hypothesis was tested on a 5% significance level. Derived from a previous study [7] we set the effect size conservatively to 0.4 and chose a sample ratio of 2:1. Based on these considerations, a sample size of 75 in the trainee group and 149 in the senior surgeon group achieved a power of 80% using two-sample t-tests (nQuery Advisor 7.0, Statistical Solutions Ltd, Cork, Ireland). From the database all patients undergoing primary TKA due to primary or secondary gonarthritis with complete pre- and postoperative outcome measures were chosen. Patients undergoing revision TKA or incomplete data files were excluded. A total of 738 patients met the inclusion criteria. All operations were performed between June 2011 and December 2015 at our Department of Orthopedic Surgery at Regensburg University Medical Center, Germany. Available data from the institutional joint registry included patient age, gender, date of admission and discharge, operative time, use of navigation, name of operating team, complications and pre- and one year postoperative Western Ontario and McMaster Universities Arthritis Index (WOMAC) [15] and Euro-Qol 5D-5L (EQ-5D) [16]. The WOMAC is an international widely used score to evaluate outcome after total joint replacement representing a multidimensional measure of pain, stiffness, and physical functional disability [17]. This measurement of outcomes by health-related quality of life questionnaire has especially been developed for patients with osteoarthritis and has been approved in several longitudinal studies with patients undergoing total joint replacement [18–20]. The EQ-5D is a widely used and tested descriptive instrument for evaluating health. It defines health based on five dimensions: Mobility, Self-Care, Usual Activities, Pain/Discomfort, and Anxiety/Depression. To improve the instrument’s sensitivity to small and medium health changes and to reduce ceiling effects the number of levels of severity in each dimension was expanded in 2005 to a five-level descriptive system increasing reliability and sensitivity of EQ-5D [16].

TKAs performed by trainees were compared to those performed by senior surgeons. Due to the prolonged operative time for navigated TKA [14], the results were separately analyzed for conventional and navigated TKAs to reduce potential bias. Therefore, all TKAs were classified into four groups: Conventional trainee TKA, conventional senior TKA, navigated trainee TKA and navigated senior TKA. TKA was defined as training procedure if the junior surgeon had completed the entire TKA. All trainee operations were performed under the supervision of a senior surgeon attending the procedure according to the national German guidelines. All trainees had a basic surgical education of 2 years prior to performing TKAs and performed their arthroplasty rotation according to the German curriculum. A total of 13 trainees performed 292 unilateral TKA´s and six senior surgeons performed 446 unilateral TKAs. Each of the senior surgeon had experience with more than 400 TKAs. Altogether 738 cases were available for analysis. Anthropometric characteristics of the study group are shown in Table 1.

**Table 1:** Anthropometric characteristics of the study group *.

N = 738	Conv Trainee	Conv Senior	Nav Trainee	Nav Senior
Number of TKAs	N = 97	N = 115	N = 195	N = 331
Age (years)	67.8 ± 9.6	67.6 ± 9.6	66.5 ± 9.5	67.7 ± 8.8
Gender (male)	31 (32.0%)	37 (32.2%)	75 (38.5%)	166 (50.2%)
ASA-Class 1	7 (7.2%)	8 (7.0%)	17 (8.7%)	39 (11.8%)
ASA-Class 2	47 (48.5%)	59 (51.3%)	103 (52.8%)	154 (46.5%)
ASA-Class 3	43 (44.3%)	47 (40.9%)	75 (38.5%)	136 (41.1%)
ASA-Class 4	0 (0.0%)	1 (0.9%)	0 (0.0%)	2 (0.6%)
Length of hospital stay (d)	9.6 ± 1.6	9.7 ± 1.9	9.5 ± 1.5	9.6 ± 1.8

* For categorical data values are given as counts and percentages, for quantitative data values are given as mean ± standard deviation.

TKA = total hip arthroplasty, Conv = conventional TKA, Nav = navigated TKA, ASA = American Society of Anesthesiologists

**Fig 1:**
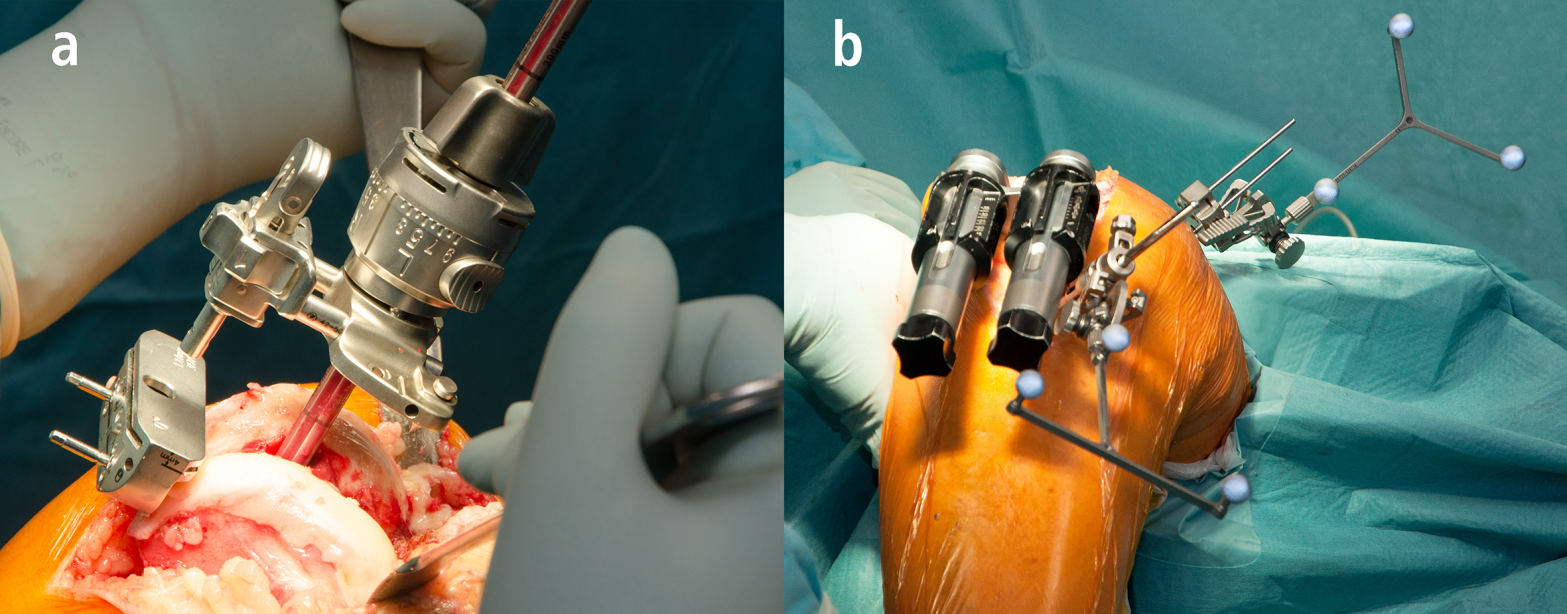
Conventional (a) and navigated (b) TKA.

An all cases, the same surgical approach and the same cemented TKA was used. Surgery was performed through a standard medial parapatellar approach including a tourniquet and a fixed bearing TKA system with cemented components of one single manufacturer (PFC Sigma^®^, DePuy,Warsaw, IN, USA). No patella resurfacing was performed. According to the senior surgeon’s preference the operation was performed with or without the use on imageless navigation device (BrainLAB, Munich, Germany). For conventional TKA, extramedullary instrumentation was used for the tibial alignment, and intramedullary alignment guides were used for the femoral component orientation. The femoral valgus angle for the intramedullary guide was determined on standardized preoperative long-leg weight bearing radiographs. For navigated TKA, after registration of bony landmarks and leg alignment the tibial resection was preformed perpendicular to the mechanical axis. Using a ligament-balanced workflow the orientation of the femoral component was determined according to the registered extension and flexion gap (Fig 1).

For dichotomizing responders and nonresponders within the first year after TKA, the Outcome Measures in Rheumatology and Osteoarthritis Research Society International consensus responder criteria (OMERACT-OARSI) were used [15, 21]. These criteria assess responder status based on relative change in Index (WOMAC) scores in relation to benchmarks determined by expert consensus and statistical analyses. OMERACT-OARSI criteria were chosen since they do not depend on patient characteristics of the cohort and thus reducing any potential selection bias due to the retrospective design of the study [22]. The OMERACT-OARSI criteria to assess responders after TKA include improvement in pain or function of at least 50% and absolute change of at least 20 points. Alternatively, responders are also defined by fulfilment of two of the following criteria: Improvement in pain of at least 20% and absolute change of at least 10 points, improvement in function of at least 20% and absolute change of at least 10 points, or global improvement of at least 20% with absolute change of at least 10 points [21].

For statistical analysis, continuous data are presented as mean ± standard deviation. Group comparisons were performed by two-sided t-tests. Counts and percentages were given for categorical data and compared between groups by chi-square tests. The primary hypothesis in the study was tested on 5% significance level. For all secondary hypotheses, significance levels were adjusted according to Bonferroni [23]. Multivariable logistic regression including age, gender, length of hospital stay, surgical experience, use of navigation, operative time, preoperative amount of pain medication and preoperative EQ-5D was performed to search for risk factors associated with responder rate within the first 12 months after TKA. IBM SPSS Statistics 22 (SPSS Inc, Chicago, IL, USA) was used for analysis.

## Results

**Fig 2:**
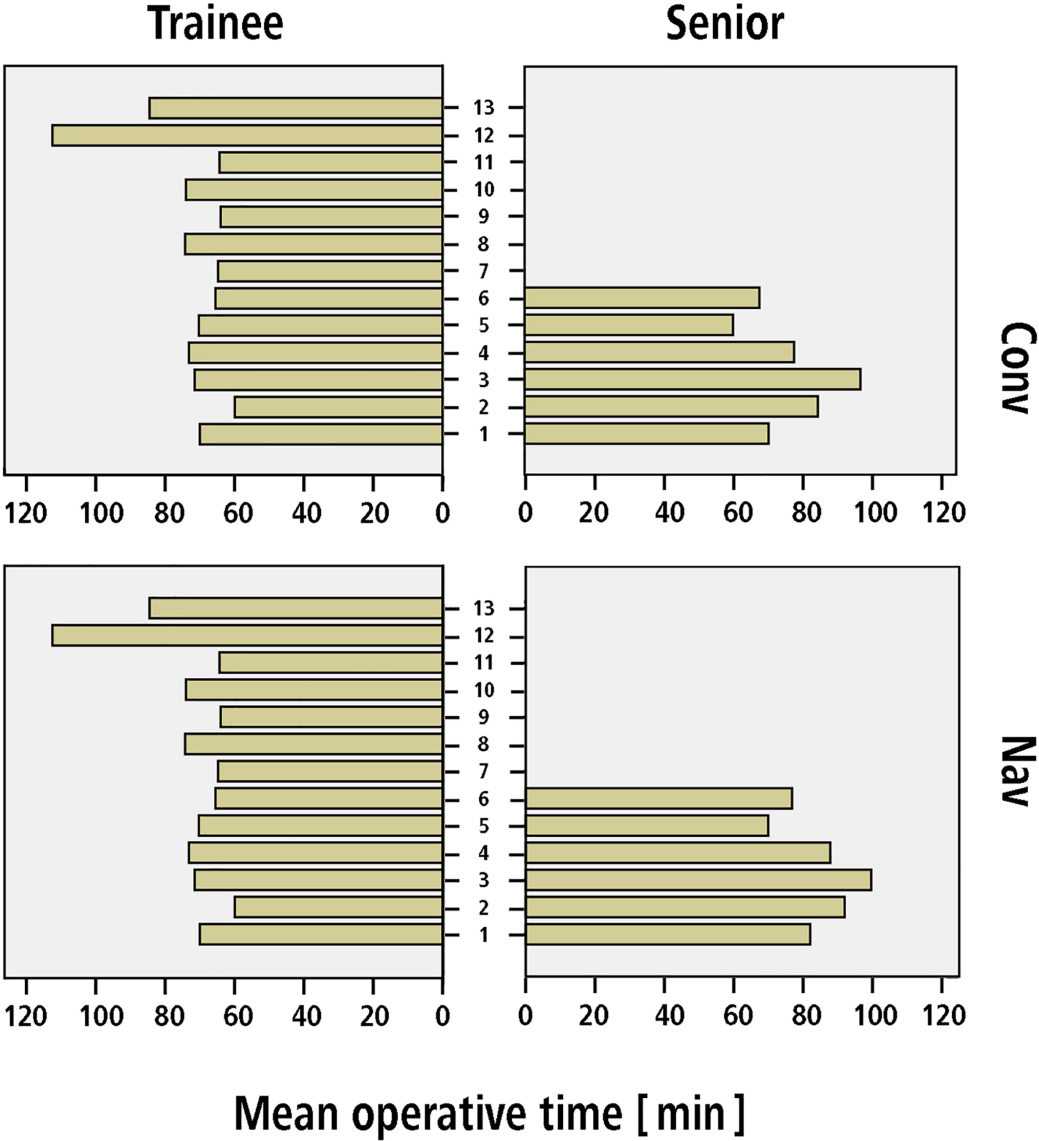
Number of TKAs performed by each surgeon (six senior surgeons and 13 trainees) during the period of the study.

Mean operative time for conventional TKA was 69.5 ± 18.5 min for trainees compared to 77.3 ± 25.8 min for senior surgeons (95% CI of the difference 1.5–13.9 min, p = 0.02). For navigated TKA, mean operative time was 80.4 ± 22.1 min for trainees compared to 84.1 ± 27.6 min for senior surgeons (95% CI of the difference -0.9–8.2 min, p = 0.12). Mean operative time for each surgeon is demonstrated in Fig 2.

Complication rates in terms of intraoperative fractures (p>0.36), postoperative thrombosis (p>0.90), postoperative neurological deficits (p>0.90) and infection rates (p>0.28) were comparable between the trainee and senior TKA groups for both conventional and navigated TKA (Table 2).

**Table 2:** Complication rates between the trainee and senior TKAs *.

**Conventional TKA**	Trainee	Senior	p-value
Intraoperative fractures	0.0% (0/97)	0.9% (1/115)	0.36
Thrombosis	1.0% (1/97)	0.9% (1/115)	0.90
Neurological deficits	1.0% (1/97)	0.9% (1/115)	0.90
Joint infection	1.0% (1/97)	0.9% (1/115)	0.90
**Navigated TKA**	Trainee	Senior	p-value
Intraoperative fractures	0.0% (0/195)	0.3% (1/331)	0.44
Thrombosis	0.0% (0/195)	0.0% (0/331)	/
Neurological deficits	0.0% (0/195)	0.0% (0/331)	/
Joint infection	0.0% (0/195)	0.6% (2/331)	0.28

* For categorical data values are given as percentages and counts

**Fig 3:**
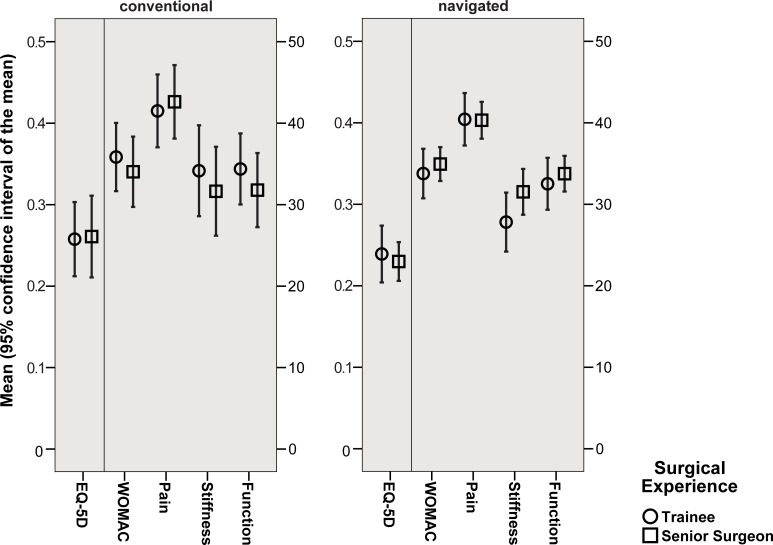
Improvement of patient reported outcome measures (WOMAC, EQ-5D) within the first year after TKA in relation to surgical experience.

Patient-reported outcome measures as assessed by WOMAC and EQ-5D showed excellent improvement within the first year postoperatively independently of trainee or senior surgeon performance (Fig 3). Analyzing outcome measures subscores, again one year results were comparable between trainee and senior TKAs (Table 3).

**Table 3:** Western Ontario and McMaster Universities Arthritis Index (WOMAC) and Euro-Qol 5D-5L (EQ-5D) by surgeon grade preoperative and 1 year after conventional (Conv) and navigated (Nav) TKA*.

**Conv TKA**		Trainee		Senior					
**Scores**		mean	SD	mean	SD	95% CI			p-value
EQ-5D	preop	0.58	0.21	0.54	0.22	-0.02	0.08		0.26
EQ-5D	postop	0.83	0.17	0.8	0.21	-0.02	0.09		0.25
WOMAC	preop	39.01	15.15	38.75	17.02	-4.14	4.67		0.91
WOMAC	postop	74.85	18.60	72.77	20.12	-3.21	7.36		0.44
Pain	preop	37.06	15.20	35.83	18.73	-3.44	5.91		0.60
Pain	postop	78.56	20.03	78.43	19.96	-5.31	5.55		0.97
Stiffness	preop	37.50	21.58	39.67	24.68	-8.51	4.16		0.50
Stiffness	postop	71.65	19.89	71.30	23.00	-5.53	6.22		0.91
Function	preop	39.77	16.53	39.50	17.9	-4.43	4.96		0.91
Function	postop	74.14	18.89	71.28	21.02	-2.60	8.31		0.30
**Nav TKA**		Trainee		Senior					
**Scores**		mean	SD	mean	SD	95% CI			p-value
EQ-5D	preop	0.56	0.21	0.59	0.20	-0.07	0.01		0.10
EQ-5D	postop	0.80	0.20	0.82	0.18	-0.05	0.01		0.23
WOMAC	preop	38.94	16.55	40.83	15.08	-4.67	0.88		0.18
WOMAC	postop	72.71	19.52	75.77	17.78	-6.33	0.21		0.07
Pain	preop	34.92	17.87	39.43	15.88	-7.45	-1.55		0.003
Pain	postop	75.36	19.91	79.74	17.91	-7.70	-1.07		0.01
Stiffness	preop	41.28	22.76	39.65	22.85	-2.42	5.68		0.43
Stiffness	postop	69.10	21.51	71.19	20.23	-5.76	1.59		0.27
Function	preop	39.84	17.32	41.39	16.18	-4.49	1.40		0.30
Function	postop	72.36	20.13	75.14	18.61	-6.18	0.62		0.11

* For quantitative data values are given as mean and SD (standard deviation).

95% CI = 95% confidence interval of the difference. preop = preoperative. postop = postoperative

For conventional TKA, the rate of responders as defined by the OMERACT-OARSI criteria [21] within the first year after TKA was similar between the trainee group with 90.7% (88/97) and the senior surgeon group 87.0% (100/115, p = 0.39). Similarly for navigated TKA, the rate of responders was comparable between trainees with 88.7% (173/195) and senior surgeons 89.4% (296/331, p = 0.80). Logistic regression analysis revealed an association between nonresponders within the first year after TKA and preoperative high EQ-5D values (OR 0.11, 95%CI 0.03–0.43, p = 0.001), whereas surgical experience had no impact on clinical outcome (Table 4).

**Table 4:** Multivariable analysis of risk factors associated with responder rate.

	OR	95% CI	P-value
Gender (male)	0.85	0.52–1.37	0.52
Age	1.00	0.98–1.03	0.83
ASA-Class	0.86	0.59–1.25	0.43
Length of hospital stay	1.03	0.89–1.19	0.71
Surgical experience (Senior)	0.97	0.60–1.58	0.90
Use of navigation	1.15	0.68–1.95	0.60
Operative time	1.00	0.99–1.01	0.38
Pain medication preop	1.00	0.99–1.01	0.95
EQ-5D preop	0.11	0.03–0.43	0.001

OR = Odds Ratio. CI = Confidence Interval. preop = preoperative, ASA = American Society of Anesthesiologists.

## Discussion

TKA is a frequently performed and successful procedure in orthopaedic surgery [1]. In order to maintain a high quality level of surgery, orthopaedic junior doctors require a structured training program with supervised operations [7, 24]. In recent years the use of navigation increased the possibility to intraoperatively control alignment and osseous resections in TKA [10]. We hypothesized that TKAs performed by orthopaedic junior doctors would (1) consume more operative time (2) be prone to higher complication rates and (3) result in lower clinical outcome within the first year after TKA when compared to senior surgeons. None of these hypotheses was supported by the results of the current study.

There are several limitations of this study. First, the study design is a retrospective, non-randomized analysis. Due to the lack of randomization, trainees might have operated on easier cases compared to senior surgeons. Therefore, the results are susceptible to potential bias. To minimize potential selection bias we chose dichotomization for responders independently of patient characteristics. Using non-cohort dependent benchmarks should maximize generalizability. Second, in one of the four subgroups (conventional TKA senior surgeons) the required sample size according to the power calculation was not achieved. However, this did not affect the robustness of our analysis since the difference in operative time between senior and trainee surgeons for conventional TKA was statistically significant. Third, the current study is restricted to the information provided by the institutional joint registry. Other parameters such as the patient’s psychological or social status might have an impact on the patient specific outcome as well. Fourth, for the current analysis only short-term outcome data for the first 12 months were available. It would have been of interest to include long-term outcome and failure rates. A strength of the study is the fact that all data refer to one single University Medical Center reflecting a specific operative workflow for conventional and navigated TKA as well as an identical postoperative treatment protocol for all patients. Similarly, an identical surgical approach and TKA components of a single manufacturer were used. All this contributes to minimizing confounding factors. Therefore, any results with regard to surgical experience is not due to surgical approach, intra- or postoperative treatment or the prosthetic component.

In answer to the first question of the study, we found no evidence of longer operative time when comparing trainees with senior surgeons for both conventional and navigated TKA. Due to previously described differences in operative time between conventional and navigated TKA [14], all analyses in this study were independently performed within the two groups to exclude this possible confounder. In contrast to our hypothesis, the mean operative time in the conventional group was not longer for trainees compared to senior surgeons. In order to reduce a potential selection bias towards easier cases for training procedures we compared distribution of ASA-classification between trainee and senior surgeons. However, the ASA scores were comparable between trainee and senior TKAs. In a previous study a 6.6 minutes (79.8 min versus 73.2 min) prolonged operative time was observed for TKA under teaching service compared to private service [7]. Another study described a 14.5 minutes longer operative time for TKAs with resident involvement (106.7 min versus 92.2 min) [8]. From an economic point of view, a prolonged operative time would mean higher financial expense for the hospital. An increase in perioperative resource consumption for TKA performed at a teaching service up to 22% has been reported in literature [25]. However, our data cannot confirm a prolonged operative time and thus extensive financial expense for TKAs performed by trainees.

In terms of our secondary outcome parameters, complications rates were similarly low in relation to other studies [7, 8]. No differences between the trainee and senior surgeon group were observed with the numbers available. This is in accordance with previous studies indicating no higher complication rates for TKAs performed by trainees [7, 8]. In contrast, a single study found an association between higher complication rates and resident participation. However, this study did not distinguish between knee and hip replacements [6]. A further study contrarily described an even lower complication rate for orthopaedic procedures with resident presence. However, this study included a variety of different orthopaedic operations and did not focus on joint replacement [9].

With the numbers available patient reported outcome measures as assessed by WOMAC and EQ-5D did not differ between the trainee and senior surgeon group. Both groups showed excellent improvement within the first year. The results are similar to previous published early results after TKA [7, 26, 27]. Accordingly responders as defined by the OMERACT-OARSI criteria [21] were not significantly different in relation to the surgical experience with a responder rate of approximately 90% in all groups. A multivariable analysis confirmed no association between responder rate and surgical intervention by trainees. Solely, high preoperative patient reported outcome measured correlated with nonresponders one year after TKA. In previous studies patients with higher preoperative pain and better preoperative function have been reported to be at high risk for worse outcome after joint replacement [28, 29].

## Conclusions

In conclusion, supervised TKA within a structured training framework is a safe procedure during the learning curve of young orthopaedic surgeons. Furthermore, the results of the current study help to reduce concerns that training in TKA might be associated with prolonged operative time, extensive financial expense, reduced patient related outcome or higher complication rates.
